# Splenectomy is associated with altered leukocyte kinetics after severe trauma

**DOI:** 10.1186/s40001-021-00497-8

**Published:** 2021-03-15

**Authors:** Michel Paul Johan Teuben, Arne Hollman, Taco Blokhuis, Roman Pfeifer, Roy Spijkerman, Henrik Teuber, Hans-Christoph Pape, Luke Petrus Hendrikus Leenen

**Affiliations:** 1 Department of Trauma, University Medical Center Utrecht, Heidelberglaan 100, 3584 CX Utrecht, The Netherlands; 2 Department of Traumatology, University Hospital Zurich, Raemistrasse 100, 8006 Zurich, Switzerland; 3 Department of Surgery, Maastricht University Medical Centre, P. Debyelaan 25, 6229 HX Maastricht, The Netherlands

**Keywords:** Trauma, Spleen, Splenectomy, Inflammation, Leukocytes

## Abstract

**Background:**

Inadequate activation of the innate immune system after trauma can lead to severe complications such as Acute Respiratory Distress Syndrome and Multiple Organ Dysfunction Syndrome. The spleen is thought to modulate the cellular immune system. Furthermore, splenectomy is associated with improved outcome in severely injured trauma patients. We hypothesized that a splenectomy alters the cellular immune response in polytrauma.

**Methods:**

All adult patients with an ISS ≥ 16 and suffering from splenic or hepatic injuries were selected from our prospective trauma database. Absolute leukocyte numbers in peripheral blood were measured. White blood cell kinetics during the first 14 days were compared between splenectomized patients, patients treated surgically for liver trauma and nonoperatively treated individuals.

**Results:**

A total of 129 patients with a mean ISS of 29 were included. Admission characteristics and leukocyte numbers were similar in all groups, except for slightly impaired hemodynamic status in patients with operatively treated liver injuries. On admission, leukocytosis occurred in all groups. During the first 24 h, leukopenia developed gradually, although significantly faster in the operatively treated patients. Thereafter, leukocyte levels normalized in all nonoperatively treated cases whereas leukocytosis persisted in operatively treated patients. This effect was significantly more prominent in splenectomized patients than all other conditions.

**Conclusions:**

This study demonstrates that surgery for intra-abdominal injuries is associated with an early drop in leucocyte numbers in peripheral blood. Moreover, splenectomy in severely injured patients is associated with an altered cellular immune response reflected by a persistent state of prominent leukocytosis after trauma.

**Supplementary Information:**

The online version contains supplementary material available at 10.1186/s40001-021-00497-8.

## Background

Trauma activates the innate immune system [1–3]. Extensive tissue damage in severe trauma evokes systemic inflammation and initiates a clinical condition known as Systematic Inflammatory Response Syndrome (SIRS) [4, 5]. Polymorphonuclear neutrophils (PMNs) are essential effector cells in inflammation in trauma. According to the SIRS-criteria, cellular immunity is altered if white blood cell (WBC) counts < 4 × 10^9^ cells/liter, total WBC count exceeds 12 × 10^9^ cells/liter, or in the case of > 10 percent immature PMNS in circulation [5].

It has been demonstrated that excessive PMN tissue influx is associated with the occurrence of early Acute Respiratory Distress Syndrome (ARDS) or Multiple Organ Dysfunction Syndrome (MODS) after trauma. [1, 6]. Excessive immune activation is also a risk factor for late septic complications after trauma [1, 7]. The inflammatory response to trauma has a bimodal pattern in which a pro-inflammatory phase is followed by an anti-inflammatory phase, however, genetic studies suggest that both pro- and anti-inflammatory pathways are activated directly after insult [8–11]. Inflammatory complications account for 50–80 percent of late mortality in surgical intensive care unit (ICU) patients. Potential interventions have been studied extensively [12, 13]. Unfortunately, no effective therapies or immunomodulatory interventions have yet been implemented [3].

According to several large database studies of severely injured patients, however, splenectomy and thereby removal of the spleens filter function is associated with improved outcome [14, 15]. Experimental studies further showed splenectomy to be associated with altered immunology, thereby underlining the immunomodulatory role of the spleen [16–23]. Splenectomy in systemic inflammation correlated with lower high mobility group box 1/Tumor necrosis factor (TNF)α-release, interleukine-6 levels and altered cellular immunity, namely impaired PMN accumulation in vital organs [16–23]. Furthermore, the clinical relevance of neutrophil responses to trauma has been demonstrated by the improved outcome after neutrophil depletion in experimental studies as well [24]. As neutrophils are the most abundant circulatory immune cells, their behavior dictates total blood leukocyte kinetics [25]. The current study aimed to investigate the impact of splenectomy on early cellular immune responses to severe trauma. We hypothesized that:

*Splenectomy is associated with altered leukocyte kinetics in peripheral blood of polytrauma patients, reflected by diminished leukopenia after insult.*

## Methods

### Patients

This study was conducted in a level one trauma center in the Netherlands (University Medical Center Utrecht). All trauma patients are registered prospectively in the institutions trauma database. We utilized this database to identify patients with blunt liver or splenic injury, between 01.01.2007 and 01.04.2015. Adults with an Injury Severity Score (ISS) ≥ 16 were included [26]. Patients transferred from other institutions and individuals who died within 24 h were excluded. Individuals with severe traumatic brain injury and subsequent poor prognosis in which withdrawal of life-sustaining measures was initiated were excluded as well. Individuals with concurrent hollow organ injuries, a combination of splenic and hepatic injuries and individuals treated by angio-embolization were excluded as well.

### Local treatment guidelines for blunt abdominal solid organ injury

Our local treatment guidelines and selection criteria for operative management (OM) are in line with international recommendations [27, 28]. In short, in our institution we tend to push nonoperative management (NOM) inclusions to the limit, and all hemodynamically stable patients with diagnosed blunt splenic or hepatic trauma that do not exhibit symptoms of hollow organ or diaphragm injury are considered as candidates for NOM. In all hemodynamically stable patients, CT scanning with intravenous contrast (and/or sonography imaging) was performed before they were admitted to the intensive care unit or ward. Angio-embolization is performed in patients initially selected for NOM and with deteriorating hemodynamic status due to diagnosed ongoing intra-abdominal blood loss. Patients with persistent hemodynamic instability, despite adequate volume and transfusion therapy are selected for exploratory laparotomy. Moreover, both guidelines and selection criteria did not change during the study period.

### Outcome parameters

*In order to test our hypothesis, and more specifically to determine the impact of a splenectomy on the systemic cellular immune response the following outcome parameters were defined and compared between groups (described hereafter).*

*Primary outcome (systemic cellular immune response):*

1. Differences in early alterations (Δ (t1-t6hours)) in circulatory leukocyte numbers between the 4 study conditions
2. Differences in circulatory leukocyte kinetics during the first 24, 48 and 72 h between the 4 study conditions
3. Differences in long-term leukocyte numbers (Δ (t1-t2weeks)) between the 4 study conditions

*Secondary outcome (clinical parameters):*

Differences in length of intensive care unit stay (ICU-LOS), hospital length of stay (H-LOS), complications and mortality.

### Study conditions

Leukocyte kinetics of the following groups were compared:

I. Blunt splenic trauma + splenectomy (OM/S)
II. Blunt splenic trauma + nonoperative management (NOM/S)
III. Blunt liver trauma + operative therapy (OM/H)
IV. Blunt liver trauma + nonoperative management (NOM/H)

### Clinical parameters

Patient and trauma characteristics as well as hemodynamic parameters including systolic blood pressure (SBP), diastolic blood pressure (DBP) and pulse rate (PR) were documented. Injury severity was determined by the Abbreviated Injury Scale of individual organ injuries and subsequent calculation of the ISS [26]. Further, data regarding all operative interventions, ICU-LOS, H-LOS, complications and mortality were collected. The following events were defined as major complications: ARDS, respiratory insufficiency, sepsis, pneumonia, urinary tract infection, abscess (abdominal/non-abdominal), wound infection, thromboembolic event, biloma, cardial decompensation, anaphylactic shock. Major inflammatory complications included: ARDS, sepsis, pneumonia, respiratory insufficiency.

### Sampling

Blood samples were routinely collected in an Ethylenediaminetetraacetic acid-coated Vacutainer. The first sample was collected on admission (t1). Thereafter, blood was drawn 6 and 16 h after admission or, in the case of a surgical intervention, after surgery. We further gathered blood samples at day 2, 3, 4, 5 or 6, 7 and after 2 weeks. Samples were analyzed by using the Cell-Dyn Sapphire hematology analyzer (Abbott Laboratories, Abbott Park, IL, USA). Blood leukocyte reference values range between 4.0–10.0 × 10^9^ cells per liter.

### Ethics

This work has been carried out in accordance with the Declaration of Helsinki and has been approved by the institution’s Medical Research Ethics Committee.

### Statistical analysis

Data were analyzed using SPSS version 22.0 software (IBM, Amsterdam, The Netherlands) and Graphpad Prism 5.0 (Graphpad software, La Jolla,San Diego, CA, USA). Results are expressed as *mean* ± *standard error of mean* (SEM) unless described otherwise. Normality of variance was tested by Lavene’s test. Student’s T test was used to analyze differences in continuous data between experimental groups with normal distributed data. In the case of discontinuous data or not normally distributed data, Mann–Whitney U tests were applied to compare groups. Categorical data were analyzed by Chi-square testing or the Fischer’s Exact test. Statistical significance was defined as *p* < 0.05.

## Results

**Fig. 1:**
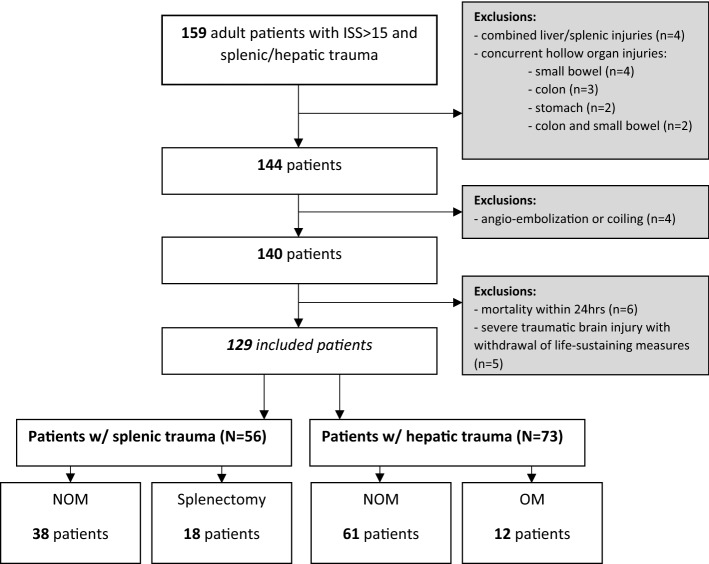
Flowchart. Absolute leukocyte numbers over time in all included trauma patients. Data are expressed as mean ± SEM. ***p < 0.001, significant alteration compared with the previous timepoint. The timepoints (in days) utilized in all further tables of the study are highlighted in grey

A total of 129 patients, of whom 92 males and 37 female patients with a mean (std) age of 34 (21–54) years were included. In- and exclusion of patients is summarized in a flowchart (Fig. 1). Forty-seven patients were injured in a motor vehicle accident, while 34 and 22 patients were injured in motorcycle and bicycle accidents, respectively. Seven patients suffered a fall from < 3 m, and 7 patients fell from a height exceeding 3 m. Three patients suffered a sport-related injury, 3 patients were injured due to violence, 2 patients got injured as a pedestrian and one person was hit by a falling object. In 3 patients the MOI was unclear (however, no signs of penetrating trauma were found).

A mean (std) Glasgow Coma Scale-score of 14 (9–15) was encountered. The mean ISS (std) of our population was 29 (22–34). The mean (std) systolic blood pressure on admission was 120 (110–137) mmHg, a mean (std) heart rate of 90 (76–110) beats per minute was documented and overall injury severity was further reflected by a mean (std) admission serum Hemoglobin level of 8.0 (7.2–8.9) g/dl.

Fifty-six patients were diagnosed with a blunt splenic injury, 18 of which were splenectomized (OM/S) while 38 were selected for nonoperative therapy (NOM/S). Seventy-three patients with blunt liver (hepatic) injury were identified. Twelve required emergency surgery (OM/H), while 61 patients were treated non-operativaly (NOM/H). In 4 out of the 12 surgically treated patients with hepatic injury, bleeding was controlled by local hemostatic techniques. In 6 individuals packing was indicated and succeeded by a planned second look. Two hemi-hepatectomies were performed, one patient underwent a cholecystectomy and two bile duct injuries were treated intra-operatively. In one patient a Pringle maneuver was executed.

A comparison of baseline parameters between groups is shown in Table 1.

**Table 1:** Baseline characteristics of all treatment groups

	Nonoperative spleen (NOM/S) *N* = *38*	Nonoperative liver (NOM/H) *N* = *61*	Splenectomy (OM/S) *N* = *18*	Operative liver (OM/H) *N* = *12*
Age (years)^a^	34 (22–55)^a^	33 (19–53)^a^	38 (21–50)^a^	39 (22–57)^a^
Gender (M/F)^a^	34/4^a^	37/24^a^	12/6^a^	9/3^a^
AIS^a^	2 (2–3)^a^	2 (2–3)^a^	4 (3–5)^a^	3 (2–4)^a^
ISS^a^	25 (20–33)	29 (21–34)^b^	29 (24–41)^a^	37 (28–47)^b^
GCS^a^	14 (9–15)^a^	14 (6–15)^a^	15 (13–15)^a^	14 (5–15)^a^
SBP (mmHg)^a^	129 (120–140)^c^	126 (113–141)^b^	110 (84–120)^c^	90 (77–120)^b^
HR (bpm)^a^	83 (68–94)^a^	90 (80–110)^a,b^	92 (75–111)^d^	100 (100–125)^b,d^
Serum Hb (mmol/L)^a^	8.7 (7.8–9.1)^c^	8.0 (7.4–8.9)^b^	7.3 (6.5–8.4)^c,d^	6.2 (4.7–7.3)^b,d^

Continuous data are presented as median (IQR) unless stated otherwise

*m* male, *f* female, *AIS* abbreviated injury scale, *ISS* injury severity score, *GCS* glasgow coma scale, *SBP* systolic blood pressure, *HR* heart rate, *Hb* hemoglobin, *bpm* beats per minute

Significance:

^a^*p* < 0.05 NOM/S vs. NOM/H

^b^*p* < 0.05 NOMH/vs. OM/H

^c^*p* < 0.05 NOM/S vs. OM/S

^d^*p* < 0.05 OM/H vs. OM/S

### Polytrauma induces a homogeneous circulatory leukocyte response

Mean admission WBC count was 15.8 ± 0.53 × 10^6^ cells/ml. Given a reference upper peak cut-off value of 10.0 × 10^6^ leukocytes/ml for homeostatic values in our laboratory, post-trauma leukocytosis was present in our trauma population. Thereafter, a statistically significant gradual drop in leukocyte numbers was observed, with the lowest values seen 24 h after trauma. Leukocyte numbers dropped to a mean minimum of 9.36 ± 0.42 × 10^6^ cells/ml, *p* < 0.01.

**Fig. 2:**
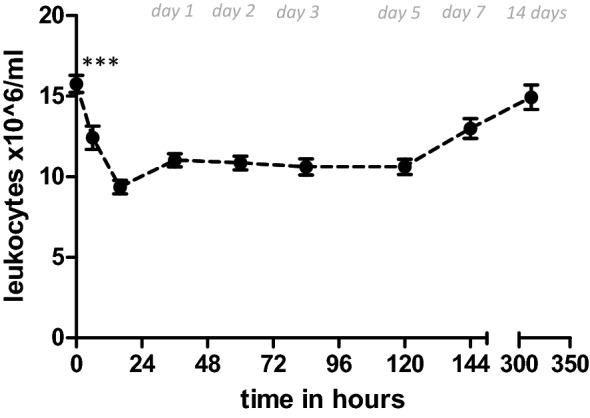
Leukocyte kinetics after blunt abdominal solid organ injury. Absolute leukocyte numbers in peripheral blood of patients treated for splenic trauma. OM, operative management group; NOM, nonoperative management group. Data are expressed as mean ± SEM. *p < 0.05/** *p* < 0.01 significant difference between groups

From the second day on, leukocyte numbers tend to further normalize and remain stable until the 5^th^ day of hospitalization. Later, white blood cell counts rise again and heterogeneity of circulatory leukocyte counts between cases increases. After 2 weeks, a mean (SEM) of 14.9 ± 0.76 × 10^6^ cells/ml was observed. Leukocyte kinetics are shown in Fig. 2.

### Splenectomy is associated with an early drop in leukocyte numbers postoperatively and a subsequent persistent leukocytosis

Admission WBC counts are similar in both the non-operative and operative splenic injury treatment groups. Directly after surgery, WBC counts were lower in the operative group vs. nonoperatively treated patients (10.0 ± 1.36 × 10^6^ L/ml vs 15.9 ± 1.66 × 10^6^ cells/ml, *p* = 0.017). Sixteen hours after trauma, leukocyte numbers in the nonoperatively treated patients normalized as well, dropping to 10.3 ± 0.68 × 10^6^ cells/ml while the WBC count in operatively treated patients fell slightly further to 8.63 ± 1.33 × 10^6^ cells/ml, *p* > 0.05.

**Fig. 3:**
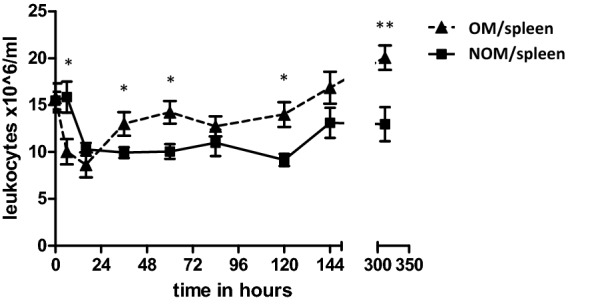
Leukocyte numbers over time in patients with splenic trauma. Absolute leukocyte numbers in peripheral blood of operatively treated patients. OM, operative management group. Data are expressed as mean ± SEM. *p < 0.05 significant difference between groups

After the first day following trauma, leukocyte numbers in splenectomized patients again increased and remained significantly elevated (with the exception of day 4) compared to nonoperatively treated patients (S/NOM). WBC counts in nonoperatively treated patients remained near homeostatic levels between day 1 and 5. From day 6 onwards, peripheral leukocyte counts showed a renewed increase in both groups, with the absolute counts remaining higher in splenectomized patients vs. the non-operative S/NOM group. Two weeks post-trauma, a striking difference between WBC counts in splenectomized patients and nonoperatively treated patients (20.07x ± 1.30 × 10^6^ L/ bml vs 12.97x ± 1.82 × 10^6^ cells/ml, *p* < 0.01) was encountered. Overall, higher leukocytes numbers were seen in spelenctomized patients compared with nonoperatively treated patients with splenic trauma. Leukocyte kinetics of patients with splenic trauma are displayed in Fig. 3.

### Surgical treatment for solid intra-abdominal organ injuries causes long-term leukocytosis, which is more prominent in splenectomized patients

To further determine the specific effect of *splenectomy* vs*. intra-abdominal surgical intervention* on leukocyte kinetics we selected patients with liver and splenic trauma. Baseline leukocyte numbers between groups were comparable at 17.1 ± 1.9 × 10^6^ L/ml in the splenic trauma group and 19.1 ± 2.17 × 10^6^ cells/ml in the liver trauma group (*p* > 0.05).

**Fig. 4:**
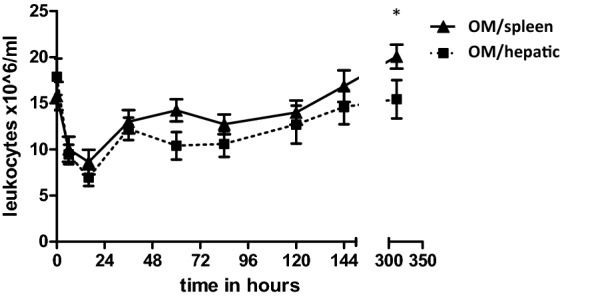
Surgical intervention and leukocyte appearance in blood. Absolute leukocyte numbers in peripheral blood of nonoperatively treated patients. NOM, non-operative management group. Data are expressed as mean ± SEM (*p < 0.05)

In both study groups, an early drop in absolute cell counts occurred directly after surgery. Minimum WBC counts in circulation were seen 16 h after trauma and were similar in both groups (*OM/S*: 7.0 ± 1.43 vs. *OM/H*: 7.0 ± 1.03 × 10^6^ cells/ml, *p* > 0.05). After the first day a similar pattern was encountered in both groups where absolute WBC counts rose gradually and peaked after two weeks. At two weeks, leukocyte numbers in patients after splenectomy were significantly higher than in the surgically treated liver trauma group (21.2 ± 1.77 × 10^6^ L/ml vs. 15.3 ± 2.34 × 10^6^ cells/ml, *p* = 0.038). Leukocyte kinetics of surgically treated patients are shown in Fig. 4.

### Leukocyte kinetics in nonoperative management of splenic and hepatic injuries are similar

**Fig. 5:**
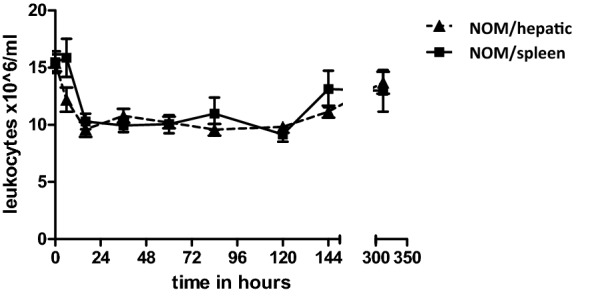
Leukocyte kinetics after intra-abdominal solid organ injury and nonoperative therapy

To determine the organ-specific effect of parenchymal tissue damage to the spleen we compared nonoperative treatment of the injured spleen with that of the injured liver. Patients treated nonoperatively for splenic and liver injuries showed similar leukocyte kinetics in peripheral blood during the 2 weeks of observation. The leukocyte pattern in the splenic and hepatic injury groups was characterized by early leukocytosis on admission (15.5 ± 0.94 × 10^6^ cells/ml vs. 15.4 ± 0.75 × 10^6^ cells/ml, *p* > 0.05). Leukocytosis resolved within 24 h with normalized WBCs. After the first-day leukocyte numbers remained within normal ranges. There was a trend toward slightly increased WBC count after two weeks, but statistical significance was not reached. Leukocytes after two weeks in non-operatively treated patients with splenic and hepatic trauma were 13.0 ± 1.82 × 10^6^ cells/ml and 13.7 ± 0.96 × 10^6^ L/ml (*p* > 0.05), respectively. Leukocyte kinetics of nonoperatively treated patients are shown in Fig. 5.

### Clinical outcome parameters: complication rates and mortality did not differ between study conditions

Patients with operatively managed liver injuries had significantly longer ICU-stays compared to all other groups (11 vs 1–3 days, *p* < 0.05). Hospital stay (31 days) in this patient group was also significantly prolonged compared to all other groups (mean stays of 14–15 days). 75 patients had an uncomplicated course and in total 89 major complications were encountered in our study. Both the type of reported complications and complication rates were similar between groups. More specifically, regarding major inflammatory complications, a total of 30 patients were diagnosed with pneumonia, sepsis occurred in 6 patients, respiratory insufficiency was seen in 12 patients. The occurrence of major inflammatory complications did not differ between study conditions. Mortality was also not significantly different between groups. Outcome parameters are summarized in Table 2.

**Table 2:** Outcome parameters in all groups

	Nonoperative Spleen (NOM/S) *N* = *38*	Nonoperative liver (NOM/H) *N* = *61*	Splenectomy (OM/S) *N* = *18*	Operative liver (OM/H) *N* = *12*
ICU-LOS median, (IQR)	2 (0–6)^b^	3 (0–10.5)^b^	1 (0–6.5)^d^	11 (4–18)^b,d^
Hospital-LOS median, (IQR)	15 (9–26)^d^	14 (7–32)^d^	14 (5–29)^d^	31 (17–46)^d^
Major complications (%)	0 (0–1) Mean: 0.56	0 (0–1) Mean: 0.45	0 (0–1) Mean: 0.50	1 (0–1.75) Mean: 1.08
Patients with uncomplicated course (%)	21 (55%)	38 (62%)	12 (72%)	4 (33%)
Mortality (%)	1 (2.6%)	3 (4.9%)	1 (5.6%)	1 (8.3%)

Data displayed as N, unless otherwise stated

*ICU* intensive care unit, *LOS* length of stay in days

Significance:

^a^*p* < 0.05 NOM/S vs. NOM/H

^b^*p* < 0.05 NOMH/vs. OM/H

^c^*p* < 0.05 NOM/S vs. OM/S

^d^*p* < 0.05 OM/H vs. OM/S

## Discussion

This study demonstrated that:

1. Surgical intervention for intra-abdominal injuries is associated with an early drop in circulatory leucocyte numbers.
2. Splenectomy in severely injured patients is associated with an altered cellular immune response compared with non-splenectomized trauma patients, and more specifically,
3. Splenectomy after splenic trauma is associated with a persistent state of prominent leukocytosis postoperatively.

This study is thereby the first to demonstrate that splenectomy in severely injured patients is associated with a modified cellular immune response. These findings suggest that the spleen plays an important immunomodulatory role in peripheral blood leukocyte homeostasis after severe trauma.

We identified a persistent state of leukocytosis in splenectomized polytrauma patients which may be the result of decreased neutrophil apoptosis as a consequence of asplenia, and subsequent prolonged survival of competent immune cells incapable of extravasating into tissue compartments [29–32]. Alternatively, asplenia may (1) stimulate an increased bone marrow release of immune cells or (2) enhance immune cell re-migration from the tissue compartment back into circulation.

Large database studies suggest that severely injured trauma patients have lower mortality rates and incidences of MODS after splenectomy [14, 15]. Various animal models of acute systemic inflammation have also shown that splenectomy is associated with improved outcome in association with the altered humoral or cellular immune response to different insult conditions [16–23]. Based on these findings and results of the current study it is tempting to speculate that early splenectomy in severely injured patients results in an altered innate cellular immune response in severely injured trauma patients and thereby influences the outcome.

The reactive leukocytosis immediately following trauma has been well described and is mainly due to an increase in circulating neutrophils [33–35]. A number of studies have shown that high neutrophil counts within the first hours after trauma is associated with increased risk of organ failure and mortality [36–38]. A key factor in the pathogenesis of MODS is the migration of blood PMNs into the tissue compartment [37, 39]. It has been shown that ARDS-related trauma fatalities were characterized by massive neutrophil influx into non-injured tissues and vital organs [40]. Circulating neutrophils are not harmful per se and peripheral blood can only function as an indicator of neutrophil behavior in the tissue compartment. So, we hypothesize that splenectomy causes shifts in neutrophil compartmentalization and the increase in circulatory leukocyte numbers seen are a result of decreased leukocyte numbers in the tissue compartment. This may explain the improved outcomes of severely injured, splenectomized patients seen in the above-mentioned studies.

The pattern of leukocyte kinetics in this human study is similar to that seen in other animal studies. For example, Eurenius et al. observed a very similar pattern in a thermal injury rat model in which early leukocytosis within the first 4 h after insult was followed by severe neutropenia [41]. The representability of our study is further supported by Botha et al. who again encountered very similar serum leukocyte patterns in laparotomized trauma patients with an ISS > 15. They demonstrated an initial increase of PMNs in peripheral blood in all patients within the first 3 h following injury. After laparotomy, neutrophil numbers normalized. Twenty-four hours after trauma, patients developed neutrophilia once again [36]. The kinetics of leukocyte alterations over time in this investigation are in line with other human trauma studies [36, 42].

Early leukocytosis, as seen on admission, in acute systemic inflammation is believed to be due to neutrophil activation in combination with mobilization of new neutrophils from the bone marrow [33–35, 43]. The observed subsequent drop in WBC count can be explained by increased tissue migration of activated neutrophils. Leukocyte numbers drop as activated neutrophils abandon the circulation and migrate into the tissue compartment. One possibility that explains why WBC counts drop faster after laparotomy is that it acts as a potent `second hit` as an additional stressor on the body and its immune system [6]. This ‘second hit’ amplifies the activation of the cellular immune system and primes blood neutrophils [2, 44]. Priming of blood neutrophils results in increased tissue migration and leukocyte depletion in peripheral blood [39]. Previous studies further showed that a faster decline in leukocyte count is associated with the development of MODS [36, 37]. Except for a slightly faster decline in leukocyte numbers after surgery, leukocyte kinetics within the first 24 h were homogeneous between groups. Overall, in our opinion neither laparotomy nor an additional splenectomy significantly alter leukocyte kinetics within the first 24 h of surgery.

More than two days after trauma, splenectomized patients had significantly higher leukocyte numbers in peripheral blood, compared with all other patient groups. Splenectomized patients again developed leukocytosis during hospital day 2 and a long-lasting leukocytosis was subsequently observed. This effect could not be explained by differences in infectious complications in asplenic patients as infectious complications did not differ between groups. Therefore, we deem this observation a result of the intrinsic effect of removing the spleen in trauma patients. This might be a direct result of the loss of splenic function as the spleen plays an important role in removing immune cells from the circulation [45]. Interestingly, neutrophil kinetics in our study were not different between patients diagnosed with pneumonia and patients without pneumonia (Additional file 1). We think that the impact of trauma and related tissue damage may have outweighed the additional effect of infection on the cellular immune response. As a consequence, we believe that monitoring leukocyte counts in splenectomized patients should be considered as suboptimal in detecting infectious complications.

The spleens modulatory role in neutrophil homeostasis has been suggested by several experimental investigations. Systemic inflammation in mice is associated with a 10-fold increase in splenic PMN-homing [46]. The characteristics and function of the splenic PMN pool is unclear. As splenic neutrophils predominantly reside in the red-pulp area of the spleen and co-localize with splenic macrophages, they are likely about to be phagocytized [47]. Thereby asplenia after trauma might result in suboptimal acute neutrophil clearance. This theory is supported by human data from Mikoluc et al. They found increased receptor membrane expression of CD16 (FcyRIII) on blood neutrophils in splenectomized children. High levels of membrane expression of CD16 are associated with phenotypical features of a more matured neutrophil population [30]. Moreover, a comparison between afferent and efferent splenic vessels suggests that the spleen promotes and modifies neutrophil apoptosis in vivo [32]. We, therefore, propose that the spleen modulates neutrophil clearance and the enhanced and long-lasting leukocytosis seen after splenectomy vs. nonoperatively managed trauma patients might be due to inadequate acute neutrophil clearance.

The importance of neutrophil apoptosis after trauma has been studied before and trauma is thought to delay PMN apoptosis [29, 31]. Surgery should be considered as iatrogenic trauma and may enhance PMN apoptosis. This phenomena might, for example, explain the slightly increased absolute leukocyte counts observed in operatively vs. non-operatively managed patients with liver injuries.

Given the fact that enhanced outcome have been observed in splenectomized trauma patients in both large database studies as well as animal studies, the spleens’ assumed role in leukocyte and neutrophil regulation, and the results of the current study, it is tempting to hypothesize that improved outcomes seen after splenectomy in polytrauma patients is due to modulation of the innate cellular immune response to trauma [14]. A possible alternative explanation is based on the splenic role in neutrophil subtype homeostasis and/or leukocyte functionality [29, 37, 42]. During systemic inflammation, Pillay et al. identified different neutrophil subtypes with specific functional capacities [48, 49]. The spleen may play a role in the modulation of these different PMN-subtypes.

In critically ill patients, mass transfusion has been linked to leukocytosis and specific systemic cytokine alterations [50, 51]. In the current study, a total of 15 patients (11.6%) received a mass transfusion. Additional sub-analysis did not show significant differences in the occurrence of early leukocytosis or persistent leukocytosis between those patients exposed to mass transfusion and regular patients (data not shown). Moreover, no correlation was found between the number of transfused PRBCs and the occurrence of systemic leukocytosis in our population of trauma patients with blunt abdominal solid organ injury (Additional file 1).

Unfortunately, in the current study we were unable to study the humoral immune response. Therefore, we could not investigate the role of a splenectomy on the interplay between the humoral immune system and the cellular immune response in patients with blunt abdominal trauma. Upcoming studies should focus on this topic as experimental data suggest a modulatory role of the spleen in both the humoral and cellular immune response to acute systemic inflammation [16–23].

A final consideration is that splenectomy may also affect the adaptive immune system. However, Walusimbi et al. found no differences in splenectomized or angio-embolized trauma patients with respect to total, helper or suppressor T-lymphocytes, complement or properdin. B-lymphocytes and natural killer cell numbers were, however, slightly higher in patients who had undergone splenectomy [52]. Additionally, Tominaga et al. could find no differences at all in serum IgM, IgG, complement and lymphocyte levels between splenectomized individuals and those treated by angio-embolization [53].

This study has several limitations. We were unable to document the time interval between the actual accident and admission to our emergency department. Nevertheless, leukocyte kinetics in our study resemble the patterns described by Botha et al. [36]. This analysis was further restricted to leukocyte measurements only. However, it has been shown that leukocyte kinetics mimic neutrophil behaviour in peripheral blood [25]. Therefore, in our opinion total leukocyte counts in peripheral blood can be utilized to indirectly assess neutrophil dynamics.

Finally, we excluded fatalities within 24 h and those patients in which withdrawal of life-saving support measures was initiated due to severe craniocerebral injuries. As a consequence, we were unable to investigate post-traumatic inflammatory neuromodulation in more detail. The interplay between cellular immune homeostasis in abdominal trauma and inflammatory neuromodulation would be an interesting topic to focus on in future projects.

In addition, the authors realize that as the grouping is based on treatment strategy there is a potential risk of selection bias. To minimize the risk of potential selection bias we decided to utilize a total of 4 study conditions. Moreover, in our institution, however, we tend to push the inclusion of candidates for NOM to the limits and this led to relatively small differences in baseline parameters between those patients selected for NOM and OM-groups. Trauma and patient characteristics including age, gender, MOI, AIS, GCS do not show clinically relevant differences, which represents no relevant selection bias. In addition, the difference in cardiopulmonary compensation between conditions in our cohort should be considered as temporarily as surgical intervention was completed upon adequate control of blood loss. And as major complications and mortality did not differ between conditions it is tempting to hypothesize that hemodynamic status on admission is not the most essential factor in outcome-prediction in our cohort. And should therefore not be considered as an essential confounding factor as well. However, more studies on this issue in blunt abdominal organ injuries are indicated.

## Conclusions

This is the first human study to demonstrate that splenectomy is associated with altered post-insult leukocyte kinetics in peripheral blood of trauma patients. These observations contribute to the assumption that the spleen plays a modulatory role in the innate cellular immune response to trauma. Moreover, these findings posit a potential explanation for the improved outcomes of splenectomized, severely injured trauma patients that has been observed in the literature. Further investigations are necessary to determine the spleens immunomodulatory role in trauma. Upcoming studies should focus on specific characteristics of leukocyte homeostasis in splenectomized and non-splenectomized trauma patients.

## Supplementary Information

**Additional file 1.** Subgroup analysis.

## Data Availability

The datasets generated and analyzed during the current study are not publicly available due to privacy limitations but are available from the corresponding author on reasonable request.

## Abbreviations

SIRS: Systematic Inflammatory Response Syndrome
PMNs: Polymorphonuclear neutrophils
WBC: White blood cell
ARDS: Acute respiratory distress syndrome
MODS: Multiple organ dysfunction syndrome
ICU: Intensive care unit
TNF: Tumor necrosis factor
ISS: Injury Severity Score
OM: Operative management
NOM: Nonoperative management
SBP: Systolic blood pressure
DBP: Diastolic blood pressure
PR: Pulse rate
ICU-LOS: Length of intensive care unit stay
H-LOS: Hospital length of stay
SEM: Standard error of mean
SD: Standard deviation

## References

[1] Keel M, Trentz O (2005). Pathophysiology of polytrauma. Injury.

[2] Hietbrink F, Koenderman L, Rijkers G, Leenen L (2006). Trauma: the role of the innate immune system. World J Emerg Surg.

[3] Lord JM, Midwinter MJ, Chen YF, Belli A, Brohi K, Kovacs EJ, Koenderman L, Kubes P, Lilford RJ (2014). The systemic immune response to trauma: an overview of pathophysiology and treatment. Lancet.

[4] Pittet D, Randel-Frausto S, Li N, Tarara D, Costigan M, Rempe L, Jebson P, Wenzel RP (1995). Systemic inflammatory response syndrome, sepsis, severe sepsis and septic shock: incidence, morbidities and outcomes in surgical ICU patients. Intensive Care Med.

[5] American College of Chest Physicians/Society of Critical Care Medicine Consensus Conference: definitions for sepsis and organ failure and guidelines for the use of innovative therapies in sepsis. Crit Care Med. 1992;20(6):864–74. 1597042

[6] Rotstein OD (2003). Modeling the two-hit hypothesis for evaluating strategies to prevent organ injury after shock/resuscitation. J Trauma.

[7] Adams JM, Hauser CJ, Livingston DH, Lavery RF, Fekete Z, Deitch EA (2001). Early trauma polymorphonuclear neutrophil responses to chemokines are associated with development of sepsis, pneumonia, and organ failure. J Trauma..

[8] Adib-Conquy M, Cavaillon JM (2009). Compensatory anti-inflammatory response syndrome. Thromb Haemost.

[9] Xiao W, Mindrinos MN, Seok J, Cuschieri J, Cuenca AG, Gao H, Hayden DL, Hennessy L, Moore EE, Minei JP, Bankey PE, Johnson JL, Sperry J, Nathens AB, Billiar TR, West MA, Brownstein BH, Mason PH, Baker HV, Finnerty CC, Jeschke MG, Lopez MC, Klein MB, Gamelli RL, Gibran NS, Arnoldo B, Xu W, Zhang Y, Calvano SE, McDonald-Smith GP, Schoenfeld DA, Story JD, Cobb JP, Warren HS, Moldawer LL, Herndon DN, Lowry SF, Maier RV, Davis RW, Tompkins RG (2011). Inflammation and Host response to injury large-scale collaborative research program. A genomic storm in critically injured humans. J Exp Med..

[10] Bone RC (1996). Sir Isaac Newton, sepsis, SIRS, and CARS. Crit Care Med.

[11] Moore FA, Sauaia A, Moore EE, Haenel JB, Burch JM, Lezotte DC (1996). Postinjury multiple organ failure: a bimodal phenomenon. J Trauma..

[12] Deitch EA (1992). Multiple organ failure. Pathophysiology and potential future therapy. Ann Surg..

[13] Roumen RM, Hendriks T, Ven-Jongekrijg J, Nieuwenhuijzen GA, Sauerwein RW, Meer JW, Goris RJ (1993). Cytokine patterns in patients after major vascular surgery, hemorrhagic shock, and severe blunt trauma. Relation with subsequent adult respiratory distress syndrome and multiple organ failure. Ann Surg..

[14] Crandall M, Shapiro MB, West MA (2009). Does splenectomy protect against immune-mediated complications in blunt trauma patients?. Mol Med.

[15] Heuer M (2010). No further incidence of sepsis after splenectomy for severe trauma: a multi-institutional experience of The trauma registry of the DGU with 1630 patients. Eur J Med Res.

[16] Huston JM, Wang H, Ochani M, Ochani K, Rosas-Ballina M, Gallowitsch-Puerta M, Ashok M, Yang L, Tracey KJ, Yang H (2008). Splenectomy protects against sepsis lethality and reduces serum HMGB1 levels. J Immunol.

[17] Huston JM, Ochani M, Rosas-Ballina M, Liao H, Ochani K, Pavlov VA, Gallowitsch-Puerta M, Ashok M, Czura CJ, Foxwell B, Tracey KJ, Ulloa L (2006). Splenectomy inactivates the cholinergic antiinflammatory pathway during lethal endotoxemia and polymicrobial sepsis. J Exp Med.

[18] Jiang H, Meng F, Li W, Tong L, Qiao H, Sun X (2007). Splenectomy ameliorates acute multiple organ damage induced by liver warm ischemia reperfusion in rats. Surgery.

[19] Hiroyoshi T, Tsuchida M, Uchiyama K, Fujikawa K, Komatsu T, Kanaoka Y, Matsuyama H (2012). Splenectomy protects the kidneys against ischemic reperfusion injury in the rat. Transpl Immunol.

[20] Chu W, Li M, Hu R, Chen Z, Lin J, Feng H (2013). Immediate splenectomy down-regulates the MAPK-NF-kappaB signaling pathway in rat brain after severe traumatic brain injury. J Trauma.

[21] Li M, Li F, Luo C, Shan Y, Zhang L, Qian Z, Zhu G, Lin J, Feng H (2011). Immediate splenectomy decreases mortality and improves cognitive function of rats after severe traumatic brain injury. J Trauma.

[22] Ito K, Ozasa H, Yoneya R, Horikawa S (2002). Splenectomy ameliorates hepatic ischemia and reperfusion injury mediated by heme oxygenase-1 induction in the rat. Liver.

[23] Okuaki Y, Miyazaki H, Zeniya M, Ishikawa T, Ohkawa Y, Tsuno S, Sakaguchi M, Hara M, Takahashi H, Toda G (1996). Splenectomy-reduced hepatic injury induced by ischemia/reperfusion in the rat. Liver.

[24] Abraham E, Carmody A, Shenkar R, Arcaroli J (2000). Neutrophils as early immunologic effectors in hemorrhage- or endotoxemia-induced acute lung injury. Am J Physiol Lung Cell Mol Physiol.

[25] Rainer TH, Chan TY, Cocks RA (1999). Do peripheral blood counts have any prognostic value following trauma?. Injury.

[26] Baker SP, Neill B, Haddon W, Long WB (1974). The injury severity score: a method for describing patients with multiple injuries and evaluating emergency care. J Trauma..

[27] Coccolini F, Montori G, Catena F, Kluger Y, Biffl W (2017). Splenic trauma: WSES classification and guidelines for adult and pediatric patients. World J Emerg Surg.

[28] Bouillon B, Marzi I (2018). The updated German "Polytrauma - Guideline": an extensive literature evaluation and treatment recommendation for the care of the critically injured patient. Eur J Trauma Emerg Surg.

[29] Biffl WL, West KE, Moore EE, Gonzalez RJ, Carnaggio R, Offner PJ, Silliman CC (2001). Neutrophil apoptosis is delayed by trauma patients' plasma via a mechanism involving proinflammatory phospholipids and protein kinase C. Surg Infect (Larchmt)..

[30] Mikoluc B, Michalkiewicz J, Motkowski R, Smolka D, Pietrucha B, Piotrowska-Jastrzebska J, Bernatowksa E (2012). Neutrophil phenotypic characteristics in children with congenital asplenia and splenectomized for hereditary spherocytosis. Immunol Invest.

[31] Nolan B, Collette H, Baker S, Duffy A, De M, Miller C, Bankey P (2000). Inhibition of neutrophil apoptosis after severe trauma is NFkappabeta dependent. J Trauma..

[32] White JF, Summers CA, Cadwallader KA, Mackenzie I, Praseedom RK, Chilvers ER, Peters AM (2011). The influence of the spleen on neutrophil apoptosis in vivo. J Cell Death.

[33] Ghebrehiwet B, Muller-Eberhard HJ (1979). C3e: an acidic fragment of human C3 with leukocytosis-inducing activity. J Immunol.

[34] Hernandez LA, Grisham MB, Twohig B, Arfors KE, Harlan JM, Granger DN (1987). Role of neutrophils in ischemia-reperfusion-induced microvascular injury. Am J Physiol.

[35] Suratt BT, Petty JM, Young SK, Malcolm KC, Lieber JG, Nick JA, Gonzalo JA, Henson PM, Worthen GS (2004). Role of the CXCR4/SDF-1 chemokine axis in circulating neutrophil homeostasis. Blood.

[36] Botha AJ, Moore FA, Moore EE, Sauaia A, Banjerjee A, Peterson VM (1995). Early neutrophil sequestration after injury: a pathogenic mechanism for multiple organ failure. J Trauma.

[37] Pallister I, Dent C, Topley N (2002). Increased neutrophil migratory activity after major trauma: a factor in the etiology of acute respiratory distress syndrome?. Crit Care Med.

[38] Visser T, Pillay J, Koenderman L, Leenen LP (2008). Postinjury immune monitoring: can multiple organ failure be predicted?. Curr Opin Crit Care.

[39] Reutershan J, Ley K (2004). Bench-to-bedside review: acute respiratory distress syndrome—how neutrophils migrate into the lung. Crit Care.

[40] Nuytinck HK, Offermans XJ, Kubat K, Goris JA (1988). Whole-body inflammation in trauma patients. An autopsy study. Arch Surg.

[41] Eurenius K, Brouse RO (1973). Granulocyte kinetics after thermal injury. Am J Clin Pathol.

[42] Ogura H, Tanaka H, Koh T, Hashiguchi N, Kuwagata Y, Hosotsubo H, Shimazu T, Sugimoto H (1999). Priming, second-hit priming, and apoptosis in leukocytes from trauma patients. J Trauma..

[43] Furze RC, Rankin SM (2008). Neutrophil mobilization and clearance in the bone marrow. Immunology.

[44] Tschoeke SK, Hellmuth M, Hostmann A, Ertel W, Oberholzer A (2007). The early second hit in trauma management augments the proinflammatory immune response to multiple injuries. J Trauma..

[45] Mebius RE, Kraal G (2005). Structure and function of the spleen. Nat Rev Immunol.

[46] Kesteman N, Vansanten G, Pajak B, Goyert SM, Moser M (2008). Injection of lipopolysaccharide induces the migration of splenic neutrophils to the T cell area of the white pulp: role of CD14 and CXC chemokines. J Leukoc Biol.

[47] Makarenkova VP, Bansal V, Matta BM, Perez LA, Ochoa JB (2006). CD11b+/Gr-1+ myeloid suppressor cells cause T cell dysfunction after traumatic stress. J Immunol.

[48] Pillay J, Kamp V, van Hoffen E, Visser T, Tak T, Lammers JW, Ulfman LH, Leenen LP, Pickkers P, Koenderman L (2012). A subset of neutrophils in human systemic inflammation inhibits T cell responses through Mac-1. J Clin Invest.

[49] Hao S, Andersen M, Yu H (2013). Detection of immune suppressive neutrophils in peripheral blood samples of cancer patients. Am J Blood Res.

[50] Izbicki G, Rudensky B, Na'amad M, Hershko C, Huerta M, Hersch M (2004). Transfusion-related leukocytosis in critically ill patients. Crit Care Med.

[51] Bogner V, Keil L, Kanz KG, Kirchhoff C, Leidel BA, Mutschler W, Biberthaler P (2009). Very early posttraumatic serum alterations are significantly associated to initial massive RBC substitution, injury severity, multiple organ failure and adverse clinical outcome in multiple injured patients. Eur J Med Res.

[52] Walusimbi MS, Dominguez KM, Sands JM, Markert RJ, McCarthy MC (2012). Circulating cellular and humoral elements of immune function following splenic arterial embolisation or splenectomy in trauma patients. Injury.

[53] Tominaga GT, Simon FJ, Dandan IS, Schaffer KB, Kraus JF, Kan M, Carlson SR, Morelands S, Nelson T, Schultz P, Eastman AB (2009). Immunologic function after splenic embolization, is there a difference?. J Trauma.

