# Supplementary material for: Splenectomy is associated with altered leukocyte kinetics after severe trauma
Source: Eur J Med Res. 2021 Mar 15;26:26. doi: 10.1186/s40001-021-00497-8 (PMC7958390; doi:10.1186/s40001-021-00497-8)
Supplement: Supplementary file 1 — Additional file 1. Subgroup analysis. [file 40001_2021_497_MOESM1_ESM.docx]

**Additional data**

**Figure S1:** Leukocyte kinetics in patients who did and did not develop pneumonia during the hospital stay


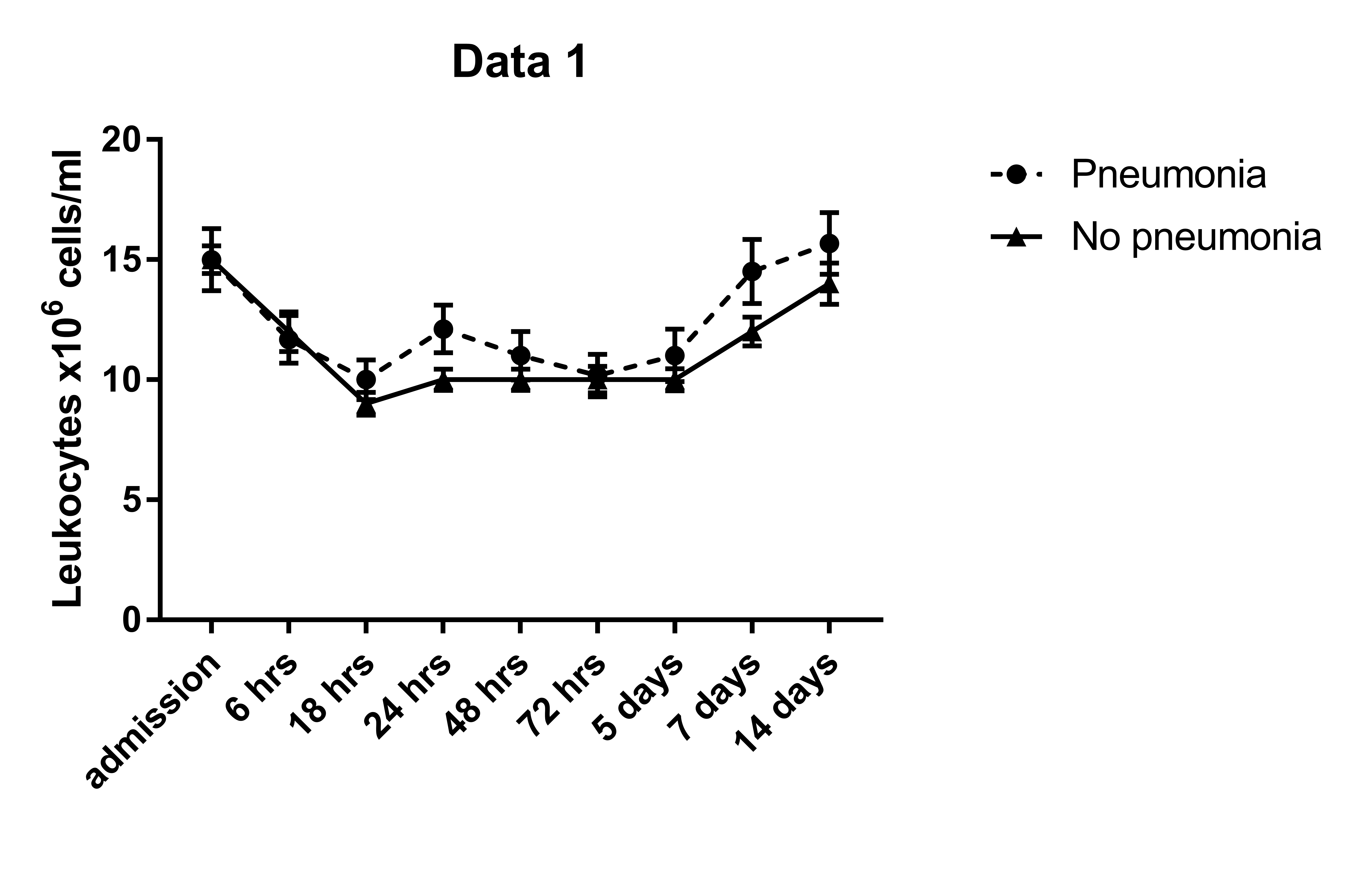


*Figure S1: Absolute leukocyte numbers in patients diagnosed with pneumonia and regular patients. Data are expressed as mean +/- SEM (*p<0.05).*

**Figure S2:** Analysis of potential correlation between the no. of transfused PRBCs and early circulatory leukocytosis


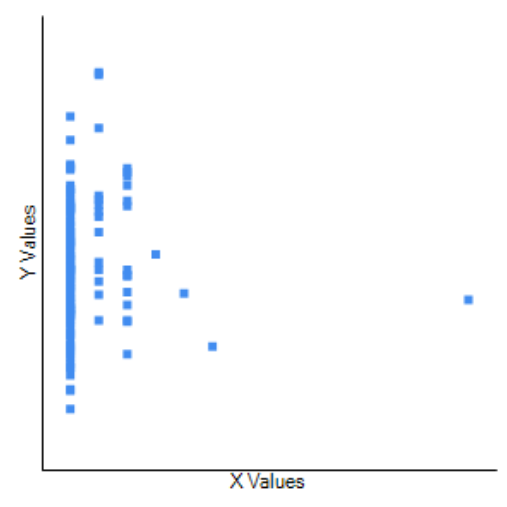


**r(127)=-0.0015, p=0.991**

*Figure S2: x-axis= no. of transfused PRBCs, y-axis= WBC_max/72hrs_ (defined as Peak-WBC-counts within 72 hours).*
